# Taxon Disappearance from Microbiome Analysis Reinforces the Value of Mock Communities as a Standard in Every Sequencing Run

**DOI:** 10.1128/mSystems.00023-18

**Published:** 2018-04-03

**Authors:** Yi-Chun Yeh, David M. Needham, Ella T. Sieradzki, Jed A. Fuhrman

**Affiliations:** a Department of Biological Sciences, University of Southern California, Los Angeles, California, USA; Northern Arizona University

**Keywords:** DNA sequencing, microbiome analysis, mock community

## Abstract

Mock communities have been used in microbiome method development to help estimate biases introduced in PCR amplification and sequencing and to optimize pipeline outputs. Nevertheless, the strong value of routine mock community analysis beyond initial method development is rarely, if ever, considered. Here we report that our routine use of mock communities as internal standards allowed us to discover highly aberrant and strong biases in the relative proportions of multiple taxa in a single Illumina HiSeqPE250 run. In this run, an important archaeal taxon virtually disappeared from all samples, and other mock community taxa showed >2-fold high or low abundance, whereas a rerun of those identical amplicons (from the same reaction tubes) on a different date yielded “normal” results. Although obvious from the strange mock community results, we could have easily missed the problem had we not used the mock communities because of natural variation of microbiomes at our site. The “normal” results were validated over four MiSeqPE300 runs and three HiSeqPE250 runs, and run-to-run variation was usually low. While validating these “normal” results, we also discovered that some mock microbial taxa had relatively modest, but consistent, differences between sequencing platforms. We strongly advise the use of mock communities in every sequencing run to distinguish potentially serious aberrations from natural variations. The mock communities should have more than just a few members and ideally at least partly represent the samples being analyzed to detect problems that show up only in some taxa and also to help validate clustering.

**IMPORTANCE** Despite the routine use of standards and blanks in virtually all chemical or physical assays and most biological studies (a kind of “control”), microbiome analysis has traditionally lacked such standards. Here we show that unexpected problems of unknown origin can occur in such sequencing runs and yield completely incorrect results that would not necessarily be detected without the use of standards. Assuming that the microbiome sequencing analysis works properly every time risks serious errors that can be detected by the use of mock communities.

## Abstract (precis)

Despite the routine use of standards and blanks in virtually all chemical or physical assays and most biological studies (a kind of “control”), microbiome analysis has traditionally lacked such standards. Here we show that unexpected problems of unknown origin can occur in such sequencing runs and yield completely incorrect results that would not necessarily be detected without the use of standards. Assuming that the microbiome sequencing analysis works properly every time risks serious errors that can be detected by the use of mock communities.

## INTRODUCTION

Analysis of microbial community composition by 16S or 18S rRNA tag sequencing is well recognized as a powerful tool to evaluate microbially diverse communities in virtually all environments (1–6). By taking advantage of high-throughput sequencing, microbial ecologists can now easily reveal microbiomes with high coverage, including the “rare biosphere.” Some potential problems are well recognized, such as chimeric sequences derived from PCR amplification artifacts (7, 8) and random sequencing errors (9, 10). A number of studies thus have developed various pipelines and algorithms to detect chimeras and other likely errors and remove them from downstream analyses (9–14). Such processing strategies help to correct or remove some errors within each sequencing run, yet they do not verify whether each sequencing and amplification analysis consistently works the same way each time and thus retrieves comparable quantitative measures of the community composition. As many microbiome projects and meta-analysis studies process sequences from multiple sequencing runs, consistency between analytical runs is critical for properly compiling data from different labs and different runs. Otherwise, the errors may be propagated in future studies.

In general, biases and many kinds of potential analytical errors can be detected by preparing and sequencing mock communities, which can act as known standards to be used during sample analysis. This is analogous to a set of standards of the sort used in virtually every careful chemical or physical assay for instrument “calibration” and/or to test that a particular batch or reagents is working as expected. Such mock communities can be genomic preparations (15) or collections of known 16S rRNA gene fragments or clones (16), which may be used for a variety of purposes but in general represent known standards. Since the abundance of operational taxonomic units (OTUs) in mock communities is known *a priori*, such mock communities can be used initially to test for biases during method development, to optimize data analysis pipelines, and also can be used in each run to verify that the analysis is within acceptable bias limits (16).

Although mock communities have been used to characterize biases and run-to-run variation in community analyses (15–18) and to support the use of highly resolving analysis approaches (19), they are still not commonly used in routine microbiome analyses. Additionally, while experimental procedures and collection are usually performed by the lab doing a given study, library preparation and/or sequencing is often performed off-site at an academic or commercial sequencing facility and to some extent in blind faith. While it may have been argued when this field was in its infancy that sequencing costs were too high to “waste” precious resources on known standards, considering the current low cost of sample preparation and analysis for sequencing, there would seem to be few excuses not to use standards today.

In this study, in contrast to the expectation that the sequencers work the same every time, we found a remarkably aberrant sequencing result, showing that an important marine taxon almost disappeared in mock communities and field samples from one sequencing run but was recovered in another run using the exact same PCR products. Other mock community taxa in the aberrant run were found at very different abundances from normal. Routine use of suitable diverse mock communities offer a good chance to detect such errors and to help validate each batch of results.

## RESULTS AND DISCUSSION

**FIG 1:**
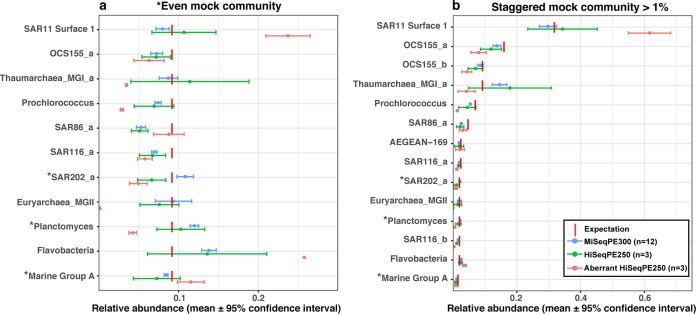
Comparisons of “even” mock communities (a) and “staggered” mock communities (b) sequenced by MiSeqPE300 and HiSeqPE250. Values that are significantly different for a clone by MiSeqPE300 versus HiSeqPE250 are indicated with an asterisk before the clone name (*P* < 0.05 by Wilcoxon rank sum test). Significant differences in the whole-community composition by MiSeqPE300 and HiSeqPE250 were found only in the even mock community (*P* < 0.05 by ANOSIM test).

We have been using mock communities for more than 4 years, primarily on the Illumina MiSeq platform, with generally consistent results from run to run (16, 19). We found mock communities particularly useful for optimizing our bioinformatic pipeline (see Materials and Methods) for the best recovery and accuracy of mock community results and with proper taxonomic assignments (16). However, we were surprised to notice that in one run analyzed on a HiSeqPE250 system (summer 2016), the mock communities yielded a completely unexpected result where the marine group II (MGII) archaea were virtually absent and other taxa had quite unexpected relative abundances whether clustered into 99% OTUs (Fig. 1) or amplicon sequence variants (ASVs) (see Fig. S1 in the supplemental material). In this single HiSeqPE250 run, we found the same result from samples prepared by three different individuals, each of whom did PCR independently and from different aliquots of the same mock community materials. This led us to reanalyze the samples and to also carefully compare several mock community runs on both the MiSeq and HiSeq platforms.

10.1128/mSystems.00023-18.1 FIG S1  Comparisons of even mock communities (a) and staggered mock communities (b) sequenced by MiSeqPE300 and HiSeqPE250 and separated into amplicon sequence variants (ASVs) by minimum entropy decomposition (14). Values that are significantly different for a clone by MiSeqPE300 versus HiSeqPE250 are indicated with an asterisk before the clone name (*P* < 0.05 by Wilcoxon rank sum test). Significant differences in the whole-community composition by MiSeqPE300 and HiSeqPE250 were found only in the even mock community (*P* < 0.05 by ANOSIM test), the same result we found by clustering into 99% OTUs (see the article). Download FIG S1, JPG file, 0.2 MB. Copyright © 2018 Yeh et al. 2018 Yeh et al. This content is distributed under the terms of the Creative Commons Attribution 4.0 International license.

An obvious first question to ask is whether this unusual result was simply due to the use of a HiSeq system rather than a MiSeq system as had been used previously. Comparison of three additional HiSeq runs to four MiSeq runs (with multiple replicates in each run) showed that there was overall run-to-run consistency (Bray-Curtis distance was 0.11 ± 0.04 in the “even” mock community and 0.12 ± 0.04 in the “staggered” mock community), with HiSeq and MiSeq producing statistically indistinguishable whole-community outcomes in “staggered” mock communities (27 taxa at different abundances), yet there were relatively modest but statistically significant differences between platforms in “even” mock communities (11 taxa at the same abundance; *P* < 0.05 and *R*^2^ = 0.47 by analysis of similarity [ANOSIM] test). When examined individually, clones in both “even” and “staggered” mock communities representing SAR202, *Planctomyces*, and marine group A, as well as OCS155_b which was included only in the staggered mock communities, were significantly different between the two sequencing platforms, in at least one comparison of OTUs (Fig. 1) or ASVs (Fig. S1) (*P* < 0.05 by Wilcoxon rank sum test). Even for the significantly different clones, the discrepancy between platforms was generally modest, with the results generally differing by less than 1.3-fold, though the SAR202 clone differed by ~1.67-fold. However, this sharply contrasted with the aberrant HiSeq sequencing run, in which mock community members representing *Euryarchaea* marine group II almost disappeared (i.e., 0 in the staggered mock community when it should have been ~1.8% and ~0.15% in the even mock community when it should have been ~9%), *Thaumarchaea* and *Prochlorococcus* were underrepresented (more than twofold) and SAR11 and Flavobacteria were overrepresented (more than twofold) (Fig. 1).

**FIG 2:**
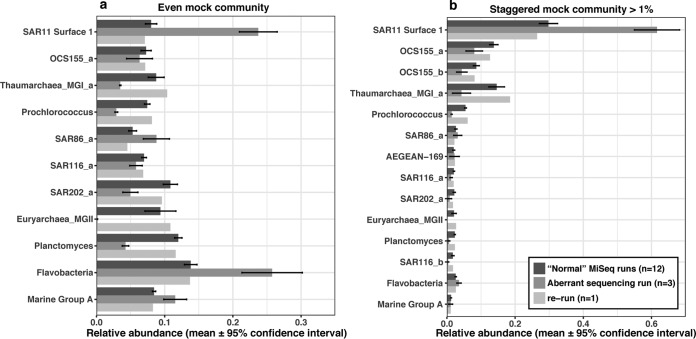
Rerun of the same PCR products (same as those shown in Fig. 1) from the “aberrant” sequencing run.

The errors responsible for the aberrant results could possibly include factors relating to (i) different proportions of sample types pooled in each run (e.g., amplicons and metagenomes), (ii) human errors and/or contamination in sample preparation, (iii) bioinformatic steps including clustering and classification, (iv) overall sequence quality and/or length, and (v) sequencing errors. We will now address these factors. (i) The samples pooled in the sequencing runs used in this study were either amplicons alone, metagenomes alone, or amplicons plus metagenomes (Table 1), but an inspection of Table 1 shows that the mixtures of sample types (from 0.1% amplicons to ~90% amplicons mixed with metagenomes) did not affect the performance of mock communities. (ii) Regarding human errors and contamination, as mentioned above, the aberrant mock community results in the single HiSeqPE300 run were found by three individuals who worked independently. The possibility of human errors in individual sample preparation seems very remote (and note that the aberrations like missing MGII archaea occurred throughout the run [see below]). Moreover, in our standard protocol, multiple blanks were included in each run to detect contamination. In the aberrant HiSeqPE300 run, the blanks were all clean with few reads, as in all the other runs. Furthermore, we do not see how contamination could possibly cause a lone taxon to disappear (including its close relatives in field samples [see below]). (iii) Bioinformatic data processing might cause problems, but to detect errors while executing complex bioinformatic steps, *in silico* sequence data were included in the processing pipeline, and the results of *in silico* mock communities showed that the pipeline performed without errors. (iv) Beyond human and systematic errors, the sequences trimmed to different lengths (for quality) could have led to less resolved OTUs, which could theoretically be problematic. However, the final trimmed sequence length of the aberrant HiSeqPE250 run was not different from normal HiSeqPE250 runs (Table 1). Furthermore, to make a systematic comparison between the MiSeqPE300 and HiSeqPE250 systems in terms of sequence length, we separately trimmed all sequences to the same length (i.e., forward reads were trimmed to 230 bases, and reverse reads were trimmed to 215 bases) before merging, and no substantial differences were found (data not shown). Moreover, the sequence error rate of each run was estimated (in mothur) by comparing sequenced mock community against the “perfect” *in silico* mock community. The results showed that the sequence error rates are all 0.02% to 0.033% (Table 1); hence, this does not explain the aberrant run either. (v) We tested whether bias was introduced in the sequencing itself (rather than in sample preparation up to and including sequencing libraries). The same PCR products of the aberrant mock communities (along with PCR products of field samples which were also included in the aberrant run) were resequenced on MiSeqPE300, and we did indeed find that sequence abundances returned to “normal,” i.e., indistinguishable from the other MiSeq runs, whether clustered by 99% OTUs (Fig. 2) or ASVs (Fig. S2). Note that with our protocols, the PCR products themselves were “ready to run” as sequencing libraries (and simply mixed with other samples that had different barcodes), so there were no additional library preparation steps that could have altered the relative compositions within samples. This indicates that the problem was related to something unique to the first sequencing run, but we cannot narrow the cause further.

10.1128/mSystems.00023-18.2 FIG S2  Rerun of the same PCR products from the “aberrant” sequencing run. The PCR products are divided into ASVs here by minimum entropy decomposition. Download FIG S2, JPG file, 0.2 MB. Copyright © 2018 Yeh et al. 2018 Yeh et al. This content is distributed under the terms of the Creative Commons Attribution 4.0 International license.

**TABLE 1:** Quality statistics of each sequencing run^a^

Sequencing platform and run	Sample types	No. of mock replicates/run^b^	Avg length of forward reads after QC^c^	Avg length of reverse reads after QC^c^	Sequence error rate (%)^d^	% sequences within expectations	*R*^2^^e^
MiSeqPE300							
Run 06	Amplicons + 10 to 15% PhiX^f^	4	286.1	261.2	0.029	98	0.94
Run 20	Amplicons + 10 to 15% PhiX^f^	3	285.6	244.7	0.03	98	0.95
Run 31	Amplicons + 10 to 15% PhiX^f^	4	278.0	221.4	0.023	99	0.95
Run 46	Amplicons + 10 to 15% PhiX^f^	1	252.7	216.9	0.029	98	0.95
**Run 40 (rerun library from the aberrant run)**	**Amplicons + 10 to 15% PhiX**^g^	**1**	**279.2**	**244.3**	**0.028**	**98**	**0.94**

HiSeqPE250							
Run 36	5% amplicons + 95% metagenomes	1	235.9	231.7	0.02	92	0.97
Run 44	Metagenomes + 0.1% mock	1	235.1	233.3	0.033	95	0.94
Run 47	20% amplicons + 80% metagenomes	1	237.5	228.2	0.018	98	0.91
**Run 37 (aberrant run)**	**20% amplicons + 80% metagenomes**	**3**	**228.4**	**240.9**	**0.021**	**97**	**0.73**^f^

a The characteristics and results for the aberrant run and the rerun of the library from the aberrant run are shown in boldface type.

b Number of mock replicates included in each run.

c The trimmed length after quality control (QC) as described in Materials and Methods.

d The error rate is defined as the sum of mismatches to the reference divided by sum of bases in query for mock communities using Mothur.

e Coefficient of variation of observed staggered mock community versus *in silico* staggered mock community under log (*x* + 0.001) at 99% similarity level.

f The *R*^2^ of the aberrant run is far outside the range of other runs.

g The sequencing facility adds 10 to 15% of PhiX174 (phage DNA) for “amplicons-only” runs as recommended by Illumina to increase sample complexity.

**FIG 3:**
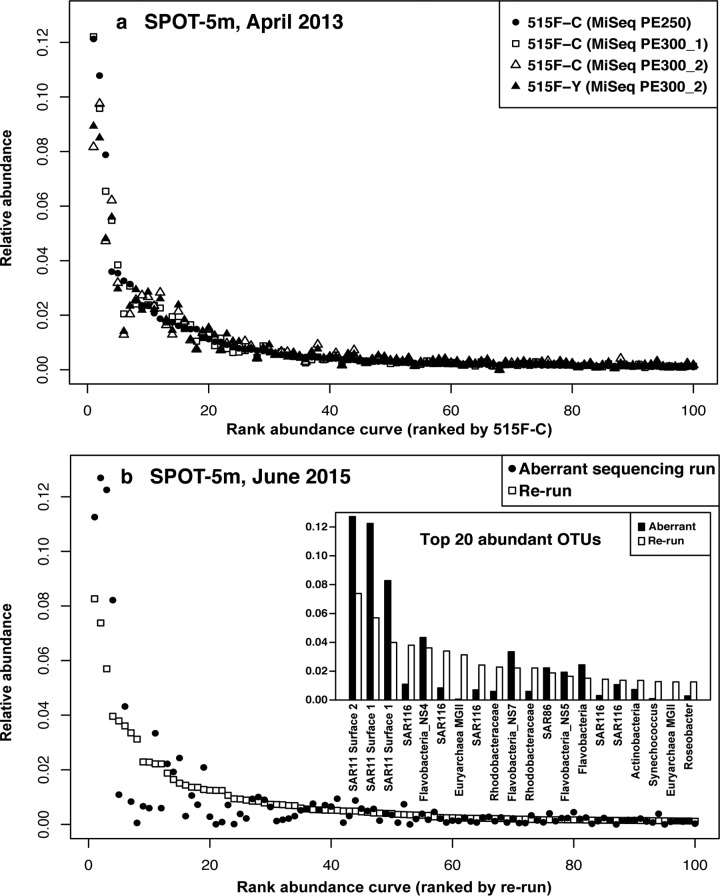
Field community comparisons via 16S and between sequencing runs. (a) Good replication of rank abundance curves between different sequencing runs and with slightly different primers (515F-C is the original EMP primer and 515F-Y is the version where a C is replaced with a Y [16]). The abundance rank was defined by primer 515F-C. (b) Comparison of rank abundance curves from the June 2015 sample analysis, showing the aberrant sequencing run and the exact same PCR products reanalyzed on the other sequencing run on a different day. The abundance rank was defined by the rerun.

To test whether the biases observed with the mock communities were also found in field samples (which would be consistent with a problem relating to the first sequencing run itself), two field samples were analyzed multiple times and compared (Fig. 3). When “normal” sequence runs were compared by analyzing a field sample (e.g., a surface seawater sample collected in April 2013 at the San Pedro Ocean Time-series [SPOT] location), the results showed that the rank abundance curves were generally similar between sequencing platforms and even between slightly different primers (Bray-Curtis distance was 0.11 ± 0.04). However, when we compared a field seawater sample collected in June 2015, which is included in the aberrant sequencing run, and resequenced the same PCR product (i.e., from the same original tube), the rank abundance curves were substantially different, considering the fact that they are supposed to be replicates (Bray-Curtis distance was ~0.31). The top 20 abundant OTUs showed that in the aberrant run SAR11 OTUs were overweighted, and *Euryarchaea* marine group II was missing—the same pattern we found in the mock communities (Fig. 1, 2, and 3b). Interestingly, in the aberrant run, three different SAR11 OTUs were strongly overrepresented, two different MGII Archaea were strongly underweighted, as were five different SAR116 taxa, suggesting that the biases were group specific (Fig. 3b). Despite these differences, which were clear by resequencing the identical sample, we note that had we not been alerted by the aberrant mock community results, the field sample results themselves did not appear so unusual overall; aside from the missing MGII archaea (which might not have been noticed or might have been thought to be real), most taxon abundances fell within the natural variation in our study area, where Bray-Curtis distances between near-surface communities typically range from ~0.2 to 0.6 (20). Hence, it was the mock community standards that revealed the problem.

### Conclusions and recommendations.

Our results suggest that including mock communities as standards in every sequencing run is strongly advised as a way to verify that each sequencing run is “behaving normally,” which we showed is not always the case. Ignoring that possibility may lead to serious errors that may obscure real patterns and lead to erroneous conclusions. Even duplicate sequencing in different runs would not help determine which data are “correct” when two runs are significantly different (and would be very expensive to do routinely). While the “missing” archaeal taxon in our study might represent a “smoking gun” of the sort that could raise concern by researchers paying close attention to each result, there could easily be less obvious changes in a given sequencing run that could strongly bias results without being noticed. In our case, because of the aberrant mock community outcomes, we were able to objectively discard the results of a run. Mock communities can also show real, even subtle, differences between analytical protocols, as we found when comparing the MiSeq and HiSeq sequencing platforms, and would be useful in revealing systematic changes introduced by any protocol modifications (planned or accidental). Because we found that only a few taxa (though common in seawater) showed the strongest biases, while others were unaffected, we suggest that mock communities should include several representative taxa to better increase the chance of detecting potential problems that may affect only some relevant taxa. Use of mock community taxa also present in the samples being analyzed is valuable for checking bioinformatic clustering (16).

Luckily, aberrant sequencing runs, in our experience, have been rare; we observed only one obviously bad one out of about 50 runs. Thus, it is reasonable to keep a running summary of results and determine whether one run is far from normal, suggesting that a reanalysis is in order. We recommend the following. (i) Ideally, each lab should develop its own baseline of expected run-to-run variation, as grounds for deciding if a given run performs “as expected.” However, before a lab has collected enough data to do that, one can start by plotting the abundance of sequenced “staggered” mock communities against the abundance of *in silico* “staggered” mock communities on a log (*x* + 0.001) scale (at 99% similarity level), allowing evaluation of the coefficient of determination (*R*^2^) of the linear regression. This is a measure of how closely the overall results meet expectation, and we think it is valuable in any case to obtain a sense of the overall quantitative accuracy of relative abundances in the community (the goal of many such studies). Note that it is normal to have some systematic biases (i.e., *R*^2^ is unlikely to be >0.97 in our experience [Table 1]), but one is looking for deviations from typical runs. While it is possible to use the *R*^2^ we report here as a starting point for what to expect and question a run if it is below ~0.85 (the *R*^2^ for our aberrant run was 0.73, and the *R*^2^ for our worst “normal” run was 0.91 [Table 1]), the *R*^2^ value may vary with lab-to-lab differences in detailed protocols, so using our results may not be applicable, and at this point, we cannot say what is “normal” in another lab. Thus, we suggest that (ii) once each lab has a baseline of multiple runs with the same methodology, compare each individual observed mock community OTU proportion over multiple runs and question the results if some OTUs in a given run are far outside the typical results. We suggest looking to see whether any taxon is >10-fold less than the average over other mock community runs (like our “disappearing” MGII archaea) or multiple taxa are >2-fold different from the average for other runs (like SAR11, *Thaumarchaea*, *Prochlorococcus*, and *Planctomyces* in Fig. 2). This can be done with “even” or “staggered” mock communities, and it is independent of expectations based on known proportions of each taxon in the mock communities. This approach is suitable with simple mock communities like our “even” mock community and when individual taxa are of particular interest. The R script of the entire analysis is available via Figshare (https://doi.org/10.6084/m9.figshare.5844075.v1). We recommend reanalyzing multiple samples from any run that appears aberrant to determine whether a systematic bias occurred throughout the run, and if there is such bias, we recommend reanalyzing all samples from that run for which quantitative data are important. If users choose to keep runs that moderately deviate from other runs, then the detection limits to judge deviations and measurement errors would increase.

## MATERIALS AND METHODS

### Sample collection and DNA extraction.

Samples were collected from a depth of 5 m at the San Pedro Ocean Time-series (SPOT) location in April 2013 and June 2016. Approximately 12 liters of seawater was prefiltered through an 80-μm mesh to remove metazoa and was then sequentially filtered through a 1.2-µm A/E filter (Pall, Port Washington, NY) and a 0.2-µm Durapore filter (ED Millipore, Billerica, MA). The filters were stored at −80°C until DNA extraction.

### Mock community preparation.

To generate even and staggered mock communities, 11 and 27 clones of marine 16S rRNA genes, respectively, were prepared (16) as follows. Briefly, clones were originally generated from 16S-ITS-23S (ITS stands for internal transcribed spacer) amplified products from marine DNA. The plasmids were purified from clones and amplified with M13F (F stands for forward) and M13R (R stands for reverse) primers. Then, bacterial 16S PCR products were generally amplified with 27F and 1492R primers, and archaeal PCR products were amplified with 20F and 1392R primers in order to obtain nearly full-length products. In the even mock community, the DNA mixture had an equal amount of each PCR product (11 in total). In the staggered mock community, the DNA mixture had different proportions of each PCR product (27 in total), roughly mimicking the marine bacterioplankton distribution from our sample site.

### PCR and sequencing.

To pool multiple samples in a single Illumina paired-end sequencing platform, a dual-index sequencing strategy was used. The V4 and V5 hypervariable regions of the 16S rRNA gene were amplified using the forward primer A-I-NNNN-barcode-515F (A-I-NNNN-barcode-GTGYCAGCMGCCGCGGTAA) and reverse primer *A*-index-*I*-926R (*A*-index-*I*-CCGYCAATTYMTTTRAGTTT), where *A* is the Illumina sequencing adapter, *I* is the Illumina primer, and barcode and index are sample-specific tags (5-bp barcode and 6-bp index). For each sample, one 25-µl amplification mixture contained 1.25× 5Prime Hot master mix (0.5 U Taq, 45 mM KCl, 2.5 mM Mg^2+^, 200 μM deoxynucleoside triphosphates [dNTPs]), 0.3 µM primers, and 0.5 ng of DNA sample. The PCR conditions were as follows: (i) an initial denaturation step of 2 min at 95°C; (ii) 30 cycles, with 1 cycle consisting of 45 s at 95°C, 45 s at 50°C, and 90 s at 68°C; and (iii) a final extension step of 5 min at 68°C. Each PCR product was cleaned using 0.8× Ampure XP magnetic beads (Beckman Coulter). Purified PCR products from samples were quantified with PicoGreen and then sequenced on Illumina HiSeq 2500 in the PE250 mode and/or MiSeq in the PE300 mode. For each sequencing run, multiple blanks and two versions of mock communities (even and staggered) were included as internal controls.

### Sequencing output processing.

Sequences were demultiplexed by reverse index allowing for one mismatch at the sequencing facility. Then, the forward barcodes were extracted using QIIME 1.9.1 *extract_barcode.py* (21). The forward and reverse reads were demultiplexed with forward barcodes independently, allowing no mismatch using QIIME 1.9.1 *split_libraires_fastq.py*. The fully demultiplexed forward and reverse reads were then split into per-sample files using QIIME *split_sequence_file_on_sample_ids.py*. The raw sequences after being demultiplexed and split into per-sample fastq files have been submitted to the EMBL database under accession numbers PRJEB12267 and PRJEB22835. The demultiplexed forward and reverse reads were quality filtered using Trimmomatic 0.36 (SLIDINGWINDOW:4:20 MINLEN:200) (22) (average lengths of the trimmed reads are shown in Table 1) and merged using USEARCH v7 *fastq_mergepairs* (23). The forward and reverse primers were then trimmed from the merged reads using cutadapt (24). Chimeric sequences were identified and removed by *de novo* chimera checking using QIIME 1.9.1 *identify_chimeric_seqs.py* and *filter_fasta.py*. Before clustering, we added to the sequences an artificial file (*in silico* expected relative abundance) containing the mock community sequences in their exact proportion and sequence composition (a “perfect” mock community) in order to help trace the outcome of the sequenced mock communities through the clustering and OTU table generation. Operational taxonomic units (OTUs) were clustered at 99% similarity cutoff by UCLUST within QIIME 1.9.1. The most abundant sequence of each OTU was chosen as the representative sequence. The taxonomy of each OTU was assigned with reference-based UCLUST against SILVA v119 database (25) using QIIME *assign_taxonomy.py*. In addition, the sequence error rate was estimated with Mothur v.1.39.5 (26) script *seq.error*. As an alternative to OTU clustering, we also implement minimum entropy decomposition (MED) (14) on our sequence set to generate amplicon sequence variants (ASVs) that differ from each other at specific bases (as distinct from OTUs that can differ at any base). In brief, MED aims to recognize real genetic variants from sequencing errors to partition the community at fine phylogenetic resolution. In our analysis, we used 0.25 as the entropy threshold to distinguish real variances from sequence errors, based on our previous work at SPOT (19). We considered only ASVs that had at least 50 individuals represented across all samples examined. Analysis by OTU clustering and MED yielded the same conclusions, and we include MED results in the supplemental material. The sequence processing script is available via FigShare (https://doi.org/10.6084/m9.figshare.5844075.v1).

### Data availability.

The processing scripts in this study are available on Figshare at https://doi.org/10.6084/m9.figshare.5844075.v1. All raw sequences have been submitted to EMBL under accession numbers PRJEB12267 and PRJEB22835.
