# Supplementary figures and images for: Taxon Disappearance from Microbiome Analysis Reinforces the Value of Mock Communities as a Standard in Every Sequencing Run
Source: mSystems. 2018 Apr 3;3(3):e00023-18. doi: 10.1128/mSystems.00023-18 (PMC5883066; doi:10.1128/mSystems.00023-18)

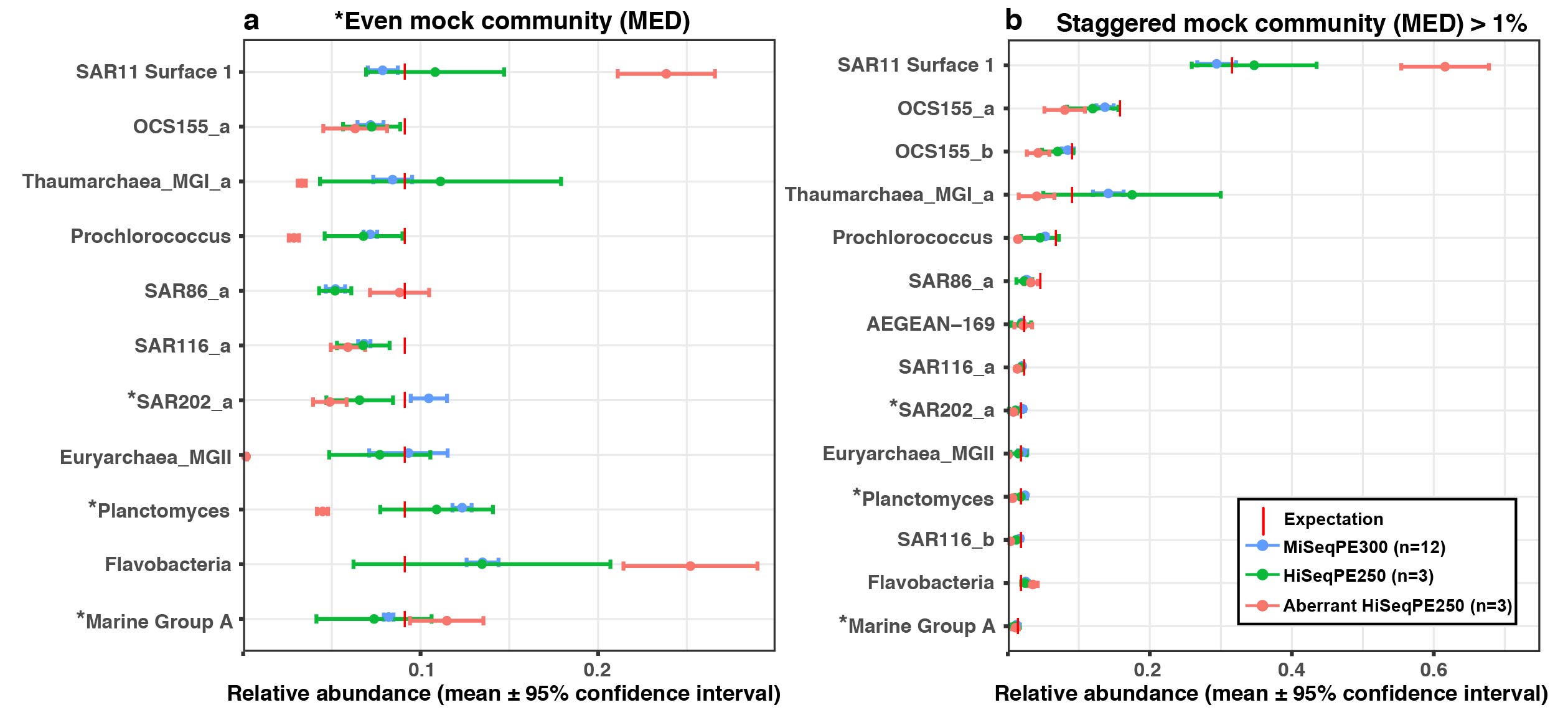

Supplement: FIG S1 [file sys003182219sf1.jpg]

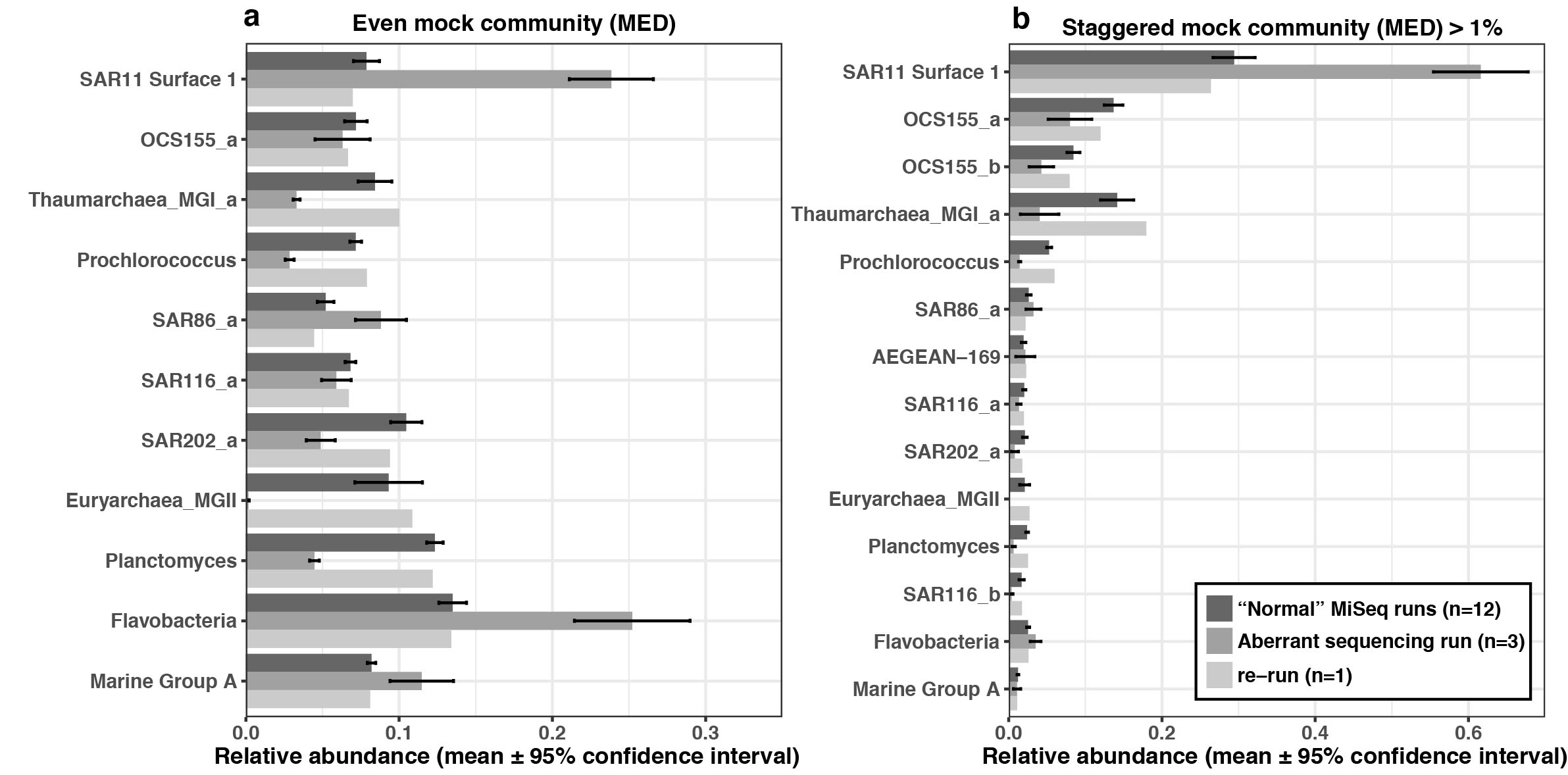

Supplement: FIG S2 [file sys003182219sf2.jpg]
